# The evolutionary landscape of chronic lymphocytic leukemia treated with ibrutinib targeted therapy

**DOI:** 10.1038/s41467-017-02329-y

**Published:** 2017-12-19

**Authors:** Dan A. Landau, Clare Sun, Daniel Rosebrock, Sarah E. M. Herman, Joshua Fein, Mariela Sivina, Chingiz Underbayev, Delong Liu, Julia Hoellenriegel, Sarangan Ravichandran, Mohammed Z. H. Farooqui, Wandi Zhang, Carrie Cibulskis, Asaf Zviran, Donna S. Neuberg, Dimitri Livitz, Ivana Bozic, Ignaty Leshchiner, Gad Getz, Jan A. Burger, Adrian Wiestner, Catherine J. Wu

**Affiliations:** 1 New York Genome Center, New York, NY 10013 USA; 2 Broad Institute, Cambridge, MA 02142 USA; 3 Meyer Cancer Center & Institute of Computational Biomedicine, Weill Cornell Medicine, New York, NY 10065 USA; 4 Hematology Branch, National Heart, Lung, and Blood Institute, National Institutes of Health, Bethesda, MD 20892 USA; 5 Sackler Medical School, Tel Aviv University, Tel Aviv, 6997801 Israel; 6 Department of Leukemia, The University of Texas MD Anderson Cancer Center, Houston, TX 77030 USA; 7 Advanced Biomedical Computing Center, Frederick National Laboratory for Cancer Research, Leidos Biomedical Research Inc, Frederick, MD 21701 USA; 8 Department of Medical Oncology, Dana-Farber Cancer Institute, Boston, MA 02215 USA; 9 Department of Biostatistics and Computational Biology, Dana-Farber Cancer Institute, Boston, MA 02215 USA; 10 Department of Applied Mathematics, University of Washington, Seattle, WA 98195 USA; 11 Harvard Medical School, Boston, MA 02215 USA

## Abstract

Treatment of chronic lymphocytic leukemia (CLL) has shifted from chemo-immunotherapy to targeted agents. To define the evolutionary dynamics induced by targeted therapy in CLL, we perform serial exome and transcriptome sequencing for 61 ibrutinib-treated CLLs. Here, we report clonal shifts (change >0.1 in clonal cancer cell fraction, *Q* < 0.1) in 31% of patients during the first year of therapy, associated with adverse outcome. We also observe transcriptional downregulation of pathways mediating energy metabolism, cell cycle, and B cell receptor signaling. Known and previously undescribed mutations in *BTK* and *PLCG2*, or uncommonly, other candidate alterations are present in seventeen subjects at the time of progression. Thus, the frequently observed clonal shifts during the early treatment period and its potential association with adverse outcome may reflect greater evolutionary capacity, heralding the emergence of drug-resistant clones.

## Abstract (web summary)

In a subset of patients with chronic lymphocytic leukemia (CLL) treated with targeted agents, such as ibrutinib, drug resistant subclones emerge. Here, the authors report on transcriptional changes in CLL patients treated with ibrutinib and identify early clonal shifts associated with evolution of resistant clones.

## Introduction

Clonal evolution is a major driving force in the ability of malignancies to adapt to therapeutic bottlenecks, becoming more aggressive and resistant to therapy. Chronic lymphocytic leukemia (CLL), the most common leukemia in western countries, illustrates the challenge posed to modern oncology by cancer evolution: despite highly effective therapies, the leukemia invariably evolves and recurs^1–4^. Indeed, in the context of standard frontline fludarabine-based chemo-immunotherapy for CLL, we have previously reported that tumor evolution following therapy is the rule rather than the exception^5,6^.

Recently, the therapeutic landscape of CLL has been dramatically altered through the introduction of multiple highly effective targeted agents^7^. Leading these is ibrutinib, a first-in-class Bruton tyrosine kinase (BTK) inhibitor, which blocks B cell receptor (BCR) signaling, a key pathway for CLL cell survival and proliferation. Ibrutinib has potent activity even in high-risk groups such as previously treated CLL or CLL with *TP53* aberrations^8–10^. Approved for all CLL patients based on improved progression free survival in treatment-naive disease and a favorable safety profile^8,11–13^, ibrutinib is increasingly used as monotherapy or tested in combination regimens.

Despite this high level of clinical activity, disease progression on ibrutinib has been increasingly appreciated, with mutations in *BTK* and in *PLCG2* (a key signaling molecule immediately downstream of BTK in the BCR pathway), as the most common adaptations to therapy^14–17^. In a limited series of CLL patients, we have also previously identified del(8p) as a putative resistance enabling driver emerging with disease progression on ibrutinib^16^. We further identified that the capacity for resistance (e.g., the observation of minute *PLCG2* mutated clones) was already present at the time of study entry, thus emphasizing the role of ibrutinib in providing strong selection pressure for the emergence of resistant clones.

Collectively, these results suggest that a study of clonal evolution patterns based on dense serial sampling and the measurement of clone-specific growth with therapy can provide clues regarding the mechanisms of resistance to this targeted agent. We therefore performed a longitudinal study of 61 CLLs treated with ibrutinib monotherapy (*n* = 45) or ibrutinib in combination with rituximab (*n* = 16), examining early patterns of clonal growth prior to development of overt CLL relapse, dynamic transcriptional changes and the course of clonal evolution upon disease progression. Here, we demonstrate that early clonal shifts, detected in 31% of patients, are associated with disease progression and identify previously undescribed mutations in *PLCG2* and *ITPKB* at the time of relapse. These data suggest that greater evolutionary capacity, as indicated by the presence of clonal shifts during the early treatment period, lead to the emergence of drug-resistant clones and an adverse clinical outcome.

## Results

### Whole-exome sequencing of samples during ibrutinib therapy

We analyzed sequential samples from 61 patients enrolled in phase 2 clinical trials of single-agent ibrutinib (NCT01500733, Cohort A, *n* = 45) or ibrutinib with rituximab (NCT02007044, Cohort B, *n* = 16). The median age of the combined cohorts was 65 (range 33–85) years and the majority of patients had markers of poor prognosis such as deletion of chromosome 17p (del(17p)) by fluorescence in situ hybridization (FISH), unmutated *IGHV* (U-*IGHV*) status and relapsed or refractory disease (Supplementary Table 1). Fifty-eight patients (95%) achieved a clinical response by 6 months. We observed marked cytoreduction across patients over the first 6 months of treatment, with a median decrease in the absolute lymphocyte count (ALC) of 64.6%, without differences between the 2 cohorts (rank sum *P* = 0.53). At a median follow-up of 48.5 months (range 5.8–58.5), 17 of 61 (28%) patients had evidence of progressive disease.

**Fig. 1:**
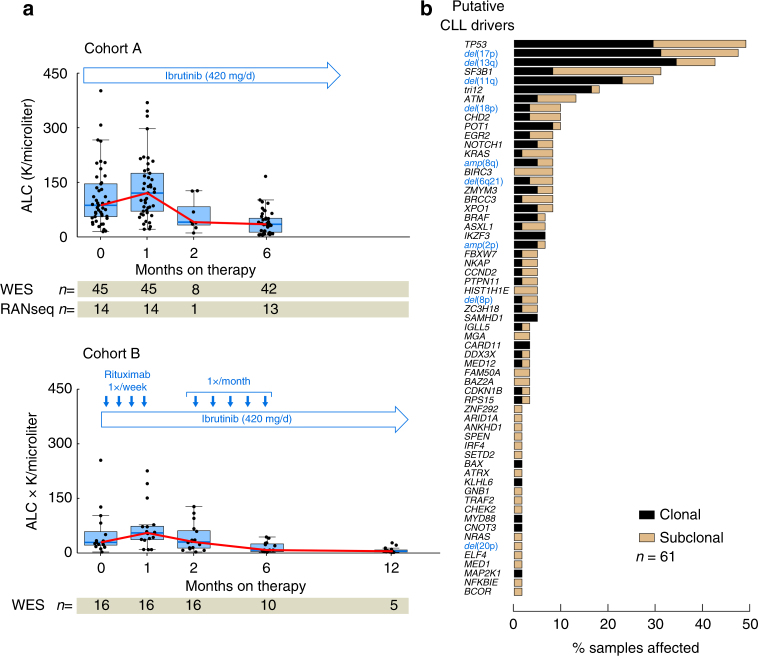
Putative driver gene mutations and copy number alterations at treatment initiation. **a** Treatment schema, absolute lymphocyte count (ALC) and number of samples per cohort that underwent whole-exome sequencing (WES) and RNA-sequencing at the indicated time points. Box plot shows the median, the interquartile range (IQR), and Tukey whiskers (±1.5 times IQR). **b** Distribution of clonal (black) and subclonal (tan) putative CLL driver mutations and copy number alterations (blue) across the 61 patients

We hypothesized that subclones within each patient may be differentially affected by ibrutinib treatment resulting in clonal shifts, and that these changes may be apparent early during therapy^18,19^. We therefore performed whole-exome sequencing (WES) on a median of 3 (range 3–5) pre-relapse peripheral blood CLL samples with median depth of coverage of ×107 (interquartile range [IQR] of 97–119X, Supplementary Data 1). Pre-study samples were available for all patients as well as samples at 1 and 6 months after therapy initiation for more than 85% of patients. Additional samples were available at 2, 3, and 12 months (Fig. 1a). For 14 patients in Cohort A, matched serial RNA-sequencing (RNA-seq) was also performed.

Consistent with previous characterizations of CLL^5^, baseline WES revealed a median mutation rate of 1.13 (IQR: 0.93–1.78) silent and non-silent somatic single nucleotide variants (sSNVs) and insertions and deletions (sIndels) per megabase across patients, with no significant differences between the two clinical cohorts (Mann–Whitney *P* = 0.156). Across the 61 pre-treatment samples, we observed 229 sSNVs and sIndels affecting 49 candidate CLL genes, and 108 CLL somatic copy number alterations (sCNAs)^6^. Of the 229 putative driver sSNVs/sIndels, 152 (66.4%) were observed at subclonal frequency^6^, while 33 of 108 (21.7%) driver sCNAs were subclonal in frequency (Fig. 1b, Supplementary Fig. 1, Supplementary Data 2 & 3). The most recurrent lesions were mutations in *TP53* and del(17p), underlining the high-risk nature of CLL patients enrolled in these early clinical studies. We note that the presence of a *TP53* alteration at baseline did not have a statistically significant association with adverse outcome (time-to-progression, log-rank *P* = 0.33). Other candidate drivers such as mutations in *SF3B1* and *ATM*, del(13q) and del(11q) were found in a similar proportion of samples, compared with our previous report^6^.

Since the BCR and nuclear factor-κB (NF-κB) pathways are directly inhibited by ibrutinib, we examined whether the presence of mutations in these pathways prior to ibrutinib therapy was associated with inferior outcome. We identified 25 pre-study samples with 32 somatic mutations in the BCR and NF-κB pathways at both clonal and subclonal frequencies (Supplementary Fig. 2a, Supplementary Data 4). Most mutations were predicted to be damaging (73.9% missense mutations with PolyPhen-2 score ≥0.95^20^) and 14 (43.8%) of 32 had been previously reported in cancer (COSMIC^21^, cancer.sanger.ac.uk, v79). We did not observe the previously described ibrutinib resistance mutations in *BTK* or *PLCG2*, although we detected a clonal *CARD11* L251P mutation reported to confer resistance to ibrutinib in diffuse large B cell lymphoma^22,23^. Despite the presence of baseline mutations in critical regulators downstream of BTK in more than a third of patients, the reduction in ALC during the first 6 months was not significantly different between patients with and without pathway mutations (rank sum *P* = 0.43). We noted only a trend towards higher risk of relapse (Fisher *P* = 0.091) and shorter time-to-progression (hazard ratio 2.1 [95% CI 0.80–5.53, log-rank *P* = 0.12, Supplementary Fig. 2b) in patients with baseline pathway mutations.

### Early clonal shifts associate with adverse clinical outcome

In addition to the baseline characterization, the frequent serial sequencing of early on-treatment (prior to relapse) samples enabled us to directly compare the relative diminution of different clonal subsets in response to ibrutinib therapy. To track clonal trajectories across serial samples, we first measured the variant allele fraction (VAF) of all sSNVs/sIndels identified in all available WES data (i.e., across the different timepoints) per patient. VAFs were transformed to cancer cell fractions (CCFs) using ABSOLUTE^24^, followed by *n*-dimensional clustering (*n* = number of timepoints per patient), across all samples. This procedure allows us to infer discrete clusters of somatic mutations that define each subclone, and to calculate the CCF of each subclone at each timepoint (Methods section).

**Fig. 2:**
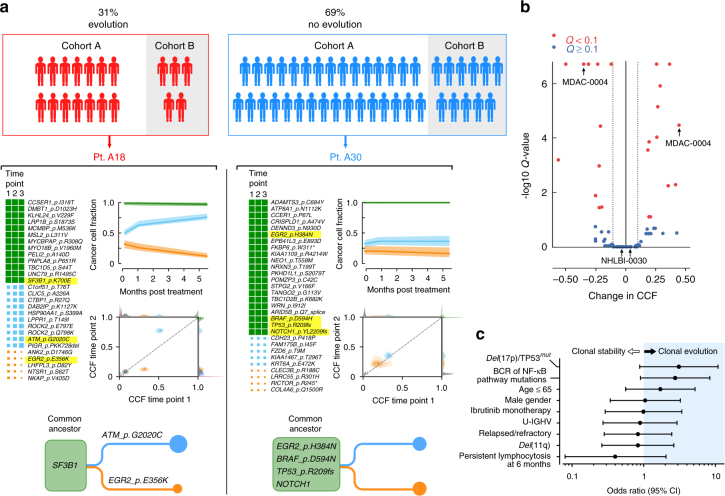
Early clonal shifts during treatment with ibrutinib. **a** 31% of CLLs showed significant clonal shifts during the first year of therapy, excluding relapse timepoints to quantify early clonal shifts in the absence of disease progression. Examples show an evolved (left) and unevolved (right) CLL. For each of the two CLLs, *n*-dimensional clustering across multiple mutations and timepoints (left panel) allows the derivation of the clonal cancer cell fraction (CCF) for each subclone at each timepoint, integrating the CCF information of each of the mutations harbored by the subclone (top panel). Comparison of clonal CCF between pre-treatment and the latest pre-relapse samples enables to determine the presence of significant clonal shifts over time (middle panel) and phylogenetic tree inference (bottom). Candidate CLL driver mutations^6^ are highlighted in yellow. **b** The magnitude of change in CCF of the largest rising or falling clone within each patient over the duration studied is displayed as a function of the CCF change. Red data points indicate significant (*q* < 0.1, see Methods section for *P* value estimation) changes in subclone size and blue indicating non-significant changes. Median values of CCF change are shown as dotted lines. **c** Association between clinical characteristics and the risk of early clonal shifts (*n* = 61, except for *U*-*IGHV* (*n* = 57) and persistent lymphocytosis at 6 months (*n* = 60) due to data availability). FISH fluorescent in situ hybridization, *U-IGHV* unmutated *IGHV* gene, CI confidence interval

For each patient, we compared the clonal composition of the baseline sample with the latest on-treatment sample, within the first 12 months of therapy (median time interval 168 days, range 56–365). Nineteen of 61 CLLs (31%) showed significant pre-relapse changes in CCF over time, defined as a FDR adjusted *P* value of <0.1 for change in CCF >0.1 in the largest rising or falling clone (Fig. 2a, Supplementary Fig. 3 & 4, see Methods for *P* value estimation). We observed a wide range of CCF changes in these clones (median 0.23 [range 0.12–0.56]; Fig. 2b). Of the 19 CLLs with significant early clonal shifts, a likely branched pattern was observed in 15 patients, in which distinct clones appeared to reciprocally rise and fall, suggestive of shifts in dominance between the two sibling clones (e.g., A18), while the remainder of evolving CLLs demonstrated a linear evolution pattern (*n* = 4), where clonal dynamics may have resulted from a change in frequency between parent and progeny clones. Neither the number of serial samples per patient (rank sum *P* = 0.3), nor the time interval between the baseline and last sample (rank sum *P* = 0.055) differed significantly between CLLs with and without clonal shifts. Moreover, the propensity for clonal shifts was not impacted by the addition of rituximab treatment (Fisher *P* = 1.0), nor by ibrutinib-related lymphocytosis, which may persist for months after therapy initiation, but has not been associated with inferior clinical outcome^11^. Indeed, the change in ALC during the first 6 months of treatment was not different between CLLs with or without early clonal shifts (rank sum *P* = 0.49). A survey of additional baseline clinical characteristics, such as *IGHV* mutation status, prior treatment history, del(17p) or *TP53* mutations, del(11q), and BCR or NF-κB pathway mutations, did not identify clear associations with early clonal shifts (Fig. 2c).

**Fig. 3:**
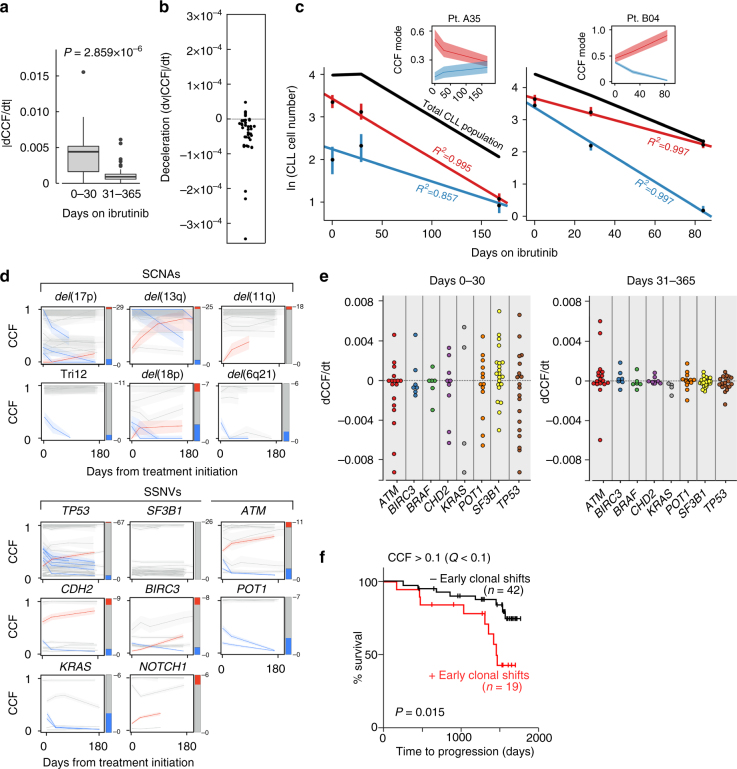
Genotype specific kinetics and disease progression on treatment. **a** Absolute CCF change per day (d|CCF|/d*t*) in the first period (first 30 days) and second period (beyond 30 days and up to 365 days) of significantly changing clones in patients with early clonal shifts. Box plot shows the median, the interquartile range (IQR), and Tukey whiskers (±1.5 times IQR, paired Welch’s *t* test). **b** The deceleration of change in CCF over the duration studied (d*v*|CCF|/d*t*) of these clones. **c** Measured circulating clonal sizes are shown for cases A35 and B04 (black dots represent the mode of CCF distribution multiplied by the number of circulating CLL cells, bars show 95% CI, CLL cell number is given in thousands of cells per microliter). The color curve represents fit with exponential growth with associated *R* ^2^ value. Total tumor burden is represented by the black line (ALC × sample purity) and insets show the corresponding changes in CCF for each CLL. **d** CCF change over time is shown for each putative CLL driver (somatic copy number alterations (SCNAs) and somatic single-nucleotide variations/indels (SSNVs)) represented by at least six instances across the patient cohorts. The bar on the right side of each plot reflects total number of clones harboring the indicated putative driver, with red, blue and gray representing the rate of significant CCF increase, decrease and no change, respectively. **e** The rate of change in CCF over time (dCCF/d*t*) does not demonstrate a clear trend by subclonal driver, as shown here in both the first time period (left) and second time period (right). **f** Kaplan–Meier plot of time-to-progression separated by the presence (red) or absence (black) of early clonal shifts (CCF change >0.1 with *Q* < 0.1)

We further leveraged our dense sampling of timepoints during ibrutinib treatment to examine the growth and decline rates of subclonal populations between any pair of timepoints. Although CCF changes were largely concordant in their directionality (78.9% (95% CI: 62.7–90.4)) throughout treatment, we observed that clonal shifts were more pronounced during rather than after the first month on treatment (e.g., *ATM*-mutated clone in A18, Fig. 2a). Thus, the median absolute change in CCF per day (d|CCF|/d*t*) of the most changing clones was 4.3 × 10^−3^ % per day (IQR 1.6–5.1 × 10^−3^ %) during the first month on ibrutinib, but 5-fold less at 8.6 × 10^−4^ % per day thereafter (IQR 5.0–12.0 × 10^−4^ %, paired Welch’s *t* test *P* < 0.000003, Fig. 3a). Consistent with this decrease in CCF change over time, the median absolute CCF change rate (d*v*|CCF|/d*t*) showed a deceleration of –2.7 × 10^−5^ per day (IQR −0.6–−5.8 × 10^−5^, Student’s one-sample *t* test *P* *<* 0.0004, Fig. 3b). CCF change deceleration was associated neither with lymphocytosis at day 30 (ANOVA *P* = 0.95) nor with the addition of rituximab (ANOVA *P* = 0.3), but was consistent with exponential decline dynamics (Fig. 3c).

CLLs that harbored a candidate CLL driver^6^ at a subclonal frequency in the baseline sample were more likely to manifest early clonal dynamics (54 vs. 27%). We examined the changes in CCF over time in those CLL drivers detected at subclonal frequency, focusing on the 14 candidate CLL genes and CNVs present in at least six cases across the 61 baseline samples. Overall, these recurrent subclonal drivers (e.g., *SF3B1* ^*mut*^, *ATM* ^*mut*^ subclones) showed clonal stability, without a clear trend towards clonal increase or clonal decrease (Fig. 3d, e). This included deletions and mutations in *TP53* (*TP53* ^*mut*^), which have been associated with adverse outcome in the setting of conventional chemotherapy. Our finding of general stability of subclones over the early treatment period, even with *TP53* ^*mut*^, is consistent with prior studies reporting comparable early response rates to ibrutinib for both *TP53* ^mut^ and *TP53* ^*wt*^ CLL^9,25^.

Despite the lack of detectable differential fitness across the individual genotypes, the presence of on-treatment clonal shifts during the first year of therapy was associated with a greater likelihood of disease progression (9 of 19 [47%] evolved CLLs vs. 8 of 42 [19%] of non-evolved CLLs, Fisher *P* = 0.032) and shorter time-to-progression (hazard ratio 3.05 [95% CI 1.05–8.91], log-rank *P* = 0.015, Fig. 3f-left). To assess the sensitivity of this finding to changes in the criterion for defining significant CCF shifts (CCF change >0.1 with FDR *Q* < 0.1), we reanalyzed the data with a less stringent criterion of CCF change greater than 0.05. While as expected, more CLLs showed clonal shifts that reached statistical significance (30/61 compared with 19/61), the shorter time-to-progression remains significant (hazard ratio 2.73 (95% CI 1.05–7.09), log-rank *P* = 0.049, Supplementary Fig. 2C). Thus, early clonal dynamics may reflect a greater evolutionary capacity, irrespective of specific driver alterations, heralding emergence of drug-resistant clones and disease progression.

### Progressive transcriptional changes during ibrutinib therapy

**Fig. 4:**
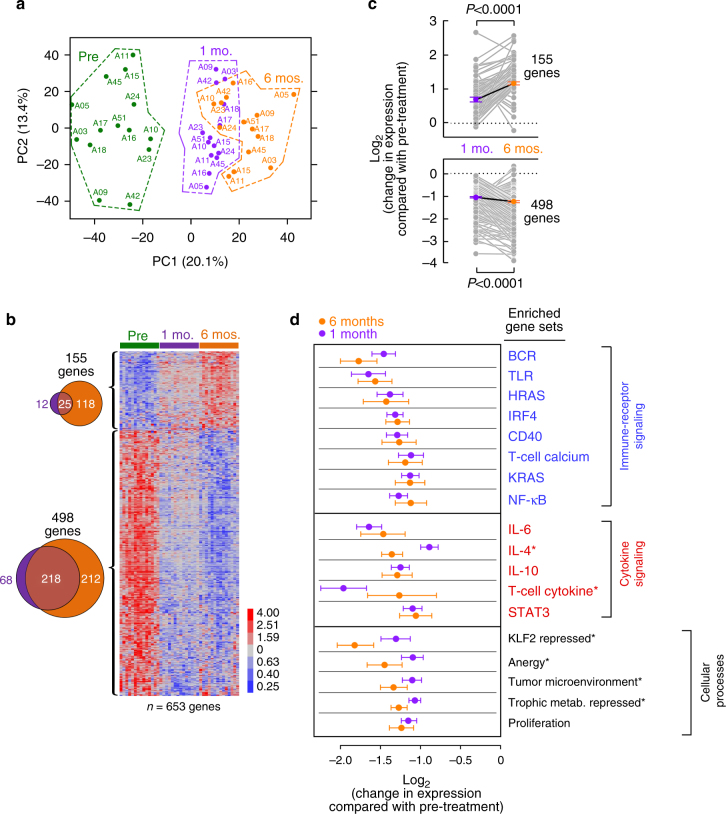
Transcriptomic changes on treatment. **a** RNA-seq principal component analysis showing PC1 and PC2 across three time points. **b** Heat map of differentially expressed genes (fold change >2; FDR < 0.1; paired Student *t* test). 155 genes increased on drug; 498 genes deceased on drug. **c** Mean (±SEM) change in gene expression compared to pre-treatment across genes upregulated (top) and downregulated (bottom) on ibrutinib therapy. *P* values determined by a paired Student *t* test **d** Mean (±SEM) change in gene expression in experimentally derived gene sets that were enriched in genes downregulated on ibrutinib across patients. Asterisks represent significant difference in gene expression between 1 month and 6 months as determined by a paired Student *t* test. Genes sets in blue represent immune-receptor signaling, red represent cytokine signaling and brown represent general cellular responses. In all panels, green color is assigned to pre-treatment samples, purple to 1 month samples and orange 6 months samples in 14 patients totaling 42 samples

To investigate the consequences of BTK inhibition on tumor cells in vivo, we sequentially profiled the CLL transcriptome in 14 patients treated with single-agent ibrutinib (Supplementary Table 1). RNA-seq was performed on CD19^+^ selected tumor cells at baseline, 1 month and 6 months on therapy. Flow cytometric analysis showed that CLL cells comprised >99% of B cells in all samples tested during the first year of therapy. After subtraction of subject-specific effects^23^ (Supplementary Fig. 5a, b) from the expression data, sampling time point became the dominant factor associated with transcriptional change, clearly separating baseline from on-treatment samples (Fig. 4a and Supplementary Fig. 5b). Of note, there was no significant correlation between the extent of treatment-induced changes in lymphocyte count and the degree of transcriptomic change (Supplementary Fig. 5c).

Comparing baseline to on-treatment samples, we identified 653 differentially expressed coding genes (fold-change ≥2 in at least one on-treatment sample, FDR < 0.1), of which 498 genes were downregulated and 155 were upregulated (Supplementary Data 5; Fig. 4b). Transcriptional changes became progressively more pronounced with treatment duration, involving a greater number of genes and increasing in magnitude of change from baseline (Supplementary Data 5; Fig. 4c). There was no difference in treatment-induced transcriptional changes between patients with early clonal shifts and those without (*P* > 0.77). Overall, these data indicate a progressively stronger impact of therapy on the transcriptome of residual tumor cells in circulation over time.

To identify specific cellular responses affected by ibrutinib, we tested for enrichment of well-characterized gene sets representing lymphocyte cellular functions and processes (Supplementary Data 6)^24,25^. Of 48 gene sets, 18 were significantly enriched with genes downregulated on ibrutinib treatment (FDR < 0.1; hypergeometric test), and followed three main categories: (i) immune-receptor signaling, (ii) cytokine signaling, and (iii) other general cellular processes (e.g., cell proliferation, Supplementary Data 6, Fig. 4d). Gene sets consistent with the first category of downregulation of immune-receptor signaling included those representing BCR (including IRF4), TLR, CD40 and canonical NF-κB signaling, consistent with the expected direct on-target effects of BTK inhibition^26^. The decrease in HRAS, KRAS, and calcium signaling likely also reflects inhibition of BCR signaling. Of the second category of cytokine signaling, and consequent downregulation of JAK/STAT signaling, we observed enrichment of gene sets representing IL-4, IL-6, IL-10, and T cell-derived cytokines, as well as those representing STAT3 expression. The third category of enriched gene sets further indicated overall reduced cell activation on ibrutinib. We observed a substantial overlap with expression changes seen in anergic B cells, as well as downregulation of genes expressed in proliferating cells, and of genes required for glucose and amino acid metabolism. This analysis is consistent with previously shown extinction of the proliferation marker Ki67 in CLL cells from patients on ibrutinib^26^, and a marked reduction in cell size by flow cytometry (Supplementary Fig. 4d, e). Finally, we found a marked reduction in genes modulated by HIF1α, a regulator of chemokine and adhesion molecules that facilitate interactions between tumor cells and the microenvironment^26^.

Overall, the transcriptomic changes on ibrutinib reflect B cells in a quiescent-like state, with substantially reduced signaling, proliferation and activation. While many of these changes are BCR-dependent, these results suggest broader effects than inhibition of BCR signaling alone. In contrast, none of the 48 gene sets were significantly enriched with genes upregulated on ibrutinib, suggesting that there is a lack of strong compensatory pathway or cellular process activation by ibrutinib.

### Relapsed disease and clonal evolution

**Fig. 5:**
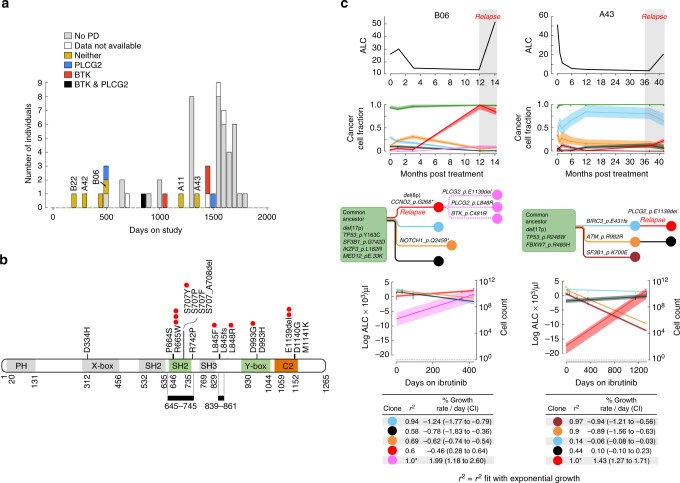
*PLCG2* mutations in ibrutinib resistant CLL. **a** The relapse characteristics are provided for the entire cohort. Patients without progressive disease (PD), are shown in gray with the time from treatment initiation to the last follow-up. For patients with PD, in addition to the time-to-progression, we provide the resistant genotype information. **b** Map of the *PLCG2* gene with mutations identified in cases of ibrutinib resistance^14,16,17,29^. Red circles denote the number of patients with indicated mutations identified in the current study. Gray bars denote the regions covered by targeted sequencing. Domains PH Pleckstrin homology, X-box phosphatidylinositol-specific phospholipase C X domain, SH2 1 C-terminal Src homology 2, SH2 2 N-terminal Src homology 2, SH3 Src homology 3, Y-box phosphatidylinositol-specific phospholipase C Y domain, C2 calcium-binding motif. **c** Detailed information is presented for the two cases in which WES revealed additional *PLCG2* mutations. Top panel shows the absolute lymphocyte count (ALC) over the patient’s clinical course, as well as changes in CCF of subclones as depicted in the inferred phylogenetic tree. Bottom panel shows the inferred growth kinetics of the different subclones, including measurements with corresponding 95% CI, as well as the exponential growth curves with 95% CI as shaded area. The calculated growth or decline rates from the exponential growth curves as well as the corresponding *R* ^2^ fit with exponential growth dynamics is listed in the table in the bottom panel. *We note that clones with *R* ^2^ = 1.0 merely reflects that only two data points were available for fitting

**Fig. 6:**
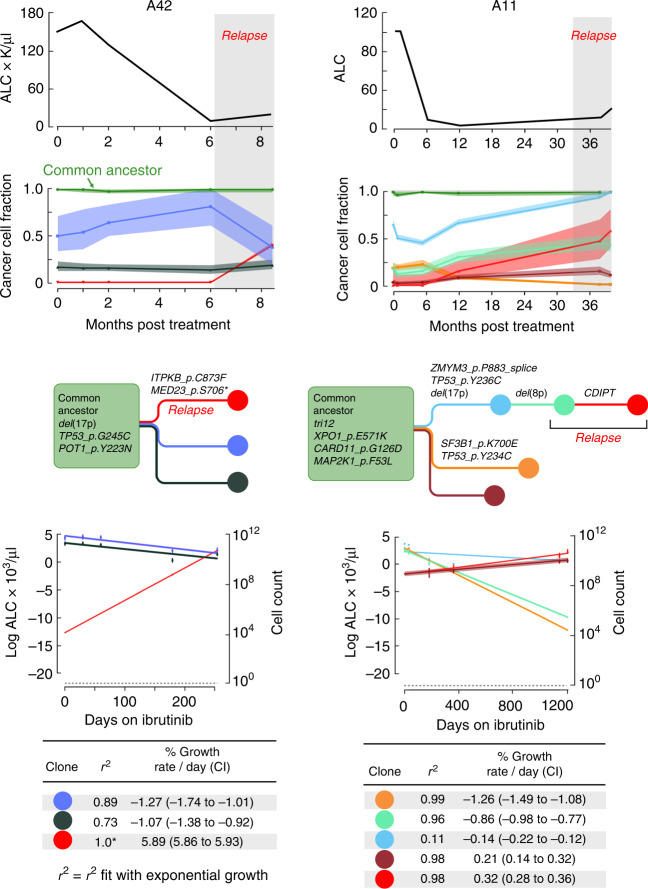
Disease progression without *BTK* or *PLCG2* mutations. As in Fig. 5, detailed information is presented for two cases in which WES revealed no *PLCG2* or *BTK* mutations. Top panel shows the absolute lymphocyte count (ALC) over the patient’s clinical course, as well as changes in CCF of subclones as depicted in the inferred phylogenetic tree. Bottom panel shows the inferred growth kinetics of the different subclones, including measurements with corresponding CI, as well as the exponential growth curves with CI as shaded area. The calculated growth or decline rates from the exponential growth curves as well as the corresponding *R* ^2^ fit with exponential growth dynamics is listed in the table in the bottom panel. *We note that *R* ^2^ = 1.0 merely reflects that only two data points were available for fitting

We have recently characterized with targeted sequencing *BTK* and *PLCG2* mutations in relapsed CLL after ibrutinib therapy^17^. We have demonstrated that these mutations may arise before the clinical appearance of relapsed disease, often with multiple clones bearing resistance variants. In the presently studied cohort, 17 of 61 patients exhibited progressive disease, 14 with relapsed CLL and 3 exhibited either transformation to aggressive lymphoma (Richter transformation: A34, A42) or to prolymphocytic leukemia (A03, Fig. 5a; Supplementary Table 2, we note that targeted sequencing was reported for 7/17 cases in Ahn et al.^17^). For 10 of 17 relapses, mutations in *BTK* and *PLCG2* were tested by targeted sequencing of known hotspots (exon 15 of *BTK*; exons 19, 20, and 24 of *PLCG2*), and detected in 6 of 10 cases (Fig. 5b, Supplementary Table 3). Based on sample availability, we undertook WES to identify putative drivers of ibrutinib resistance in four relapse cases without detectable *BTK/PLCG2* mutations by targeted sequencing (from Cohort A) or that did not undergo targeted sequencing (from Cohort B, Fig. 5c, Fig. 6).

In two of four relapse cases, we identified previously undescribed mutations in *PLCG2*. Patient B06 experienced CLL relapse 12 months following combined ibrutinib and rituximab, with a rise to dominance of a subclone harboring del(8p), which we previously linked to ibrutinib resistance^16^. This growing CLL clone yielded additional progeny clone(s) with a CCF of 0.12 at relapse, containing the canonical C481R *BTK* resistance variant, and two additional previously unreported *PLCG2* variants (L848R with a high Polyphen-2^20^ score of 0.993 for a damaging mutation, and an in-frame three base pair deletion in codon E1139del). Although the resolution of our data led our clustering algorithm to assign these three mutations to a single subclone, these likely represent three distinct sibling subclones based on our previous single-cell sequencing analysis of a similar case (Fig. 5c)^16^. Patient A43 exhibited an early rise in CCF following initiation of therapy of a subclone with *BIRC3* mutation, which was later slowly replaced by the time of relapse at 42 months by a progeny clone carrying the same E1139 deletion in the C2 terminal domain of the *PLCG2* gene. This region was outside of the territory assayed by the targeted sequencing assay, is involved in calcium binding and membrane anchoring^27,28^, and appears to be a hotspot for resistant mutations along with *PLCG2* D1140G and M1141K, previously described in ibrutinib-treated patients with progressive CLL^16,29^.

Two of the four relapse cases studied with WES did not harbor *BTK* or *PLCG2* mutations. In Patient A11, relapse occurred at 39 months and was driven by a progeny of a del(8p)-clone, containing a single Indel on the gene *CDIPT* (Fig. 6). Mutations in this gene have not been previously reported in CLL, and thus this Indel may reflect a passenger event with potential additional (e.g., non-coding or non-genetic) changes within this subpopulation that have led to its accelerated growth in the presence of ibrutinib. This patient also had an ancestral gain-of-function *CARD11* G126D mutation at baseline that has been shown to activate the NF-κB pathway in transfected HEK293 cells^30^. However, the early response and marked clonal shifts with relapse suggest that this mutation was not sufficient for ibrutinib resistance in a CLL context. Patient A42 exhibited Richter’s transformation after 9 months of therapy driven by a CLL-related clone with an *ITPKB* somatic substitution (C873F, PolyPhen-2 score 0.986, Fig. 6). We have previously reported this gene as recurrently affected in CLL^5^, and frequent alterations have also been observed in diffuse large B cell lymphoma^31^. ITPKB is a central feedback inhibitor of the BCR pathway, and its disruption may provide enhanced BCR signaling downstream of BTK^32^.

From CCF information across serial measurements, we calculated clone-specific growth rates, based on exponential growth models, which are well supported by experimental CLL data^16,33^. For most relapse clones, we found growth rates of 0.1–2% per day (e.g., *PLCG2* E1139del in Patient A43 (1.43% per day, 95% CI: 1.27–1.71%)), consistent with previous estimates of relapse-driving clones in CLL^16,33^. As expected, the clone with *ITPKB* mutation in the Richter’s transformation (Patient A42) showed a much higher growth rate of 5.89% per day (95% CI: 5.86–5.93%). In some cases, the exponential growth dynamics of the likely resistant clone were insufficient to explain the resurgence of the CLL. For example, in Patient A43, in addition to the rise of the mutant *PLCG2* clone, another large clone (*BIRC3* mutant) that was previously declining in cell number, registered an increase in number in the peripheral blood upon relapse. This suggests that either additional genetic (not covered with WES) or non-genetic (e.g., cell–cell interaction, epigenetic) factors are contributing to the relapse.

We back-extrapolated the calculated growth rates to estimate of the size of the resistant clone at treatment initiation. These analyses suggested that the del(8p) clone of Patient B06 involved 4 × 10^9^ circulating cells at treatment initiation (95% CI 2.8–5.5 × 10^9^ cells), while the emerging progeny *BTK/PLCG2* clones involved only 2.5 × 10^6^ cells at baseline (95% CI: 0.26–19 × 10^6^ cells). Likewise, the *PLCG2* mutated clone in Patient A43 appeared to be present at the time of treatment initiation at a much smaller size with a predicted cell number of only 75 cells (95% CI 12–514). Similarly, the *ITPKB* mutated Richter’s transformation clone was estimated to involve a small proportion of circulating cells at treatment initiation, estimated at 10,700 cells (95% CI 10,000–11,400).

## Discussion

The potent therapeutic efficacy of ibrutinib results in major clinical benefit for patients with CLL. However, this therapy also exerts strong selective pressure, which can promote the eventual outgrowth of resistant subclones. We therefore undertook an effort to perform unbiased sequencing of serially collected samples in order to define the evolutionary dynamics of ibrutinib-treated CLL, as we and others have shown that closely timed temporal sampling of tumor samples is a powerful approach to characterize such changes^16,18,19^.

Our data support the presence of marked clonal shifts within the early, pre-relapse treatment period in nearly a third of these high-risk ibrutinib-treated CLLs. While clonal dynamics were common, no single common CLL driver (e.g., *SF3B1* ^*mut*^ or *ATM* ^*mut*^) exhibited a clear trend of clonal expansion suggestive of selective fitness to ibrutinib. Furthermore, mutations in BCR signaling components, such as *CARD11* L251P-conferring ibrutinib refractoriness in DLBCL, did not promote clonal fitness or preclude clinical response to ibrutinib in CLL. Likewise, in contrast to the patterns observed with fludarabine-based therapy, *TP53* ^*mut*^ subclones had similar likelihood to increase or decrease on treatment, arguing against a direct relationship between *TP53* ^*mut*^ and clonal fitness in the context of ibrutinib therapy. Instead, we speculate that the genomic instability in *TP53* ^*mut*^ cancer may potentially lead to greater clonal diversity and a higher chance of acquiring additional resistance mutations. This is consistent with the clinical observation that *TP53* ^*mut*^ CLLs show similar ibrutinib-response dynamics to *TP53* ^*wt*^ CLL^9^, but may be more likely to relapse. Thus, early clonal dynamics may serve as an indicator of the evolutionary capacity of the disease, which may underlie the potential association between early clonal dynamics and adverse outcome.

Our strategy of using serial sampling may also enhance our appreciation of dynamics of transcriptional changes in response to ibrutinib. Ibrutinib induced widespread changes in gene expression in CLL cells in vivo, reflecting strong inhibitory effects across multiple pathways. This suggests a broader impact on CLL biology than expected from the loss of BTK function alone. Moreover, we did not identify any distinct biologic signatures that were upregulated in response to ibrutinib, which may indicate the absence of a cellular stress response. This is consistent with the apparent resting state of residual circulating CLL cells and their slow attrition over time. Importantly, we observed reinforcement of this transcriptional response with time, which was unrelated to clonal shifts or the degree of decrease in cell number. This may be related to the fact that ibrutinib not only affects tumor cells directly, but also leads to widespread changes in the tumor microenvironment as reported by us and others, including a decrease in inflammatory cytokines, shifts in T cell subsets and interactions between tumor cells and macrophages^34,35^. The dynamic transcriptional changes observed in this study suggest that these phenomena may involve long-term remodeling, in addition to the immediate effects of ibrutinib therapy on CLL cells.

This study also offered an opportunity to better characterize the genetic landscape of relapsed CLL after ibrutinib therapy. Our results confirm previous studies that highlight mutations in *BTK* and *PLCG2* as central to ibrutinib resistance^14–16^. By employing WES, we further identify additional putative sites within the *PLCG2* gene, including a recurrent small deletion in the C2 terminal domain found in two relapse cases. Thus, our analysis informs cases of progressive disease in which targeted sequencing was unable to explain resistance. These results suggest that clinical investigations of ibrutinib resistance may benefit from comprehensive sequencing of *PLCG2* until the spectrum of resistance mutations is fully characterized. As previously observed^14,16,17^, relapsed CLL may often contain multiple distinct mutations in these resistance genes highlighting the tremendous evolutionary capacity of this disease. The estimation of the size of resistant clones, based on the back-extrapolation of growth rates, showed that typically resistance arises from pre-existing clonal diversity^36,37^. We note that these calculations rely on the assumption of exponential growth with stable growth rates, an assumption we have previously validated experimentally^16^, but remains to be further tested. Finally, it is also important to consider that the characterization of the resistance genotypes in CLL is likely incomplete. Here, we identify an additional candidate relapse driver—*ITPKB*. *ITPKB* mutations are significantly overrepresented in DLBCL^38,39^, and in *ITPKB* ^−/−^ mice, B cell lymphomas develop with constitutive activation of PI3K signaling^40^. This example suggests that efforts to study relapse cases should continue as our experience with ibrutinib therapy broadens, especially in these relapses without known *PLCG2* or *BTK* mutations.

Collectively, these findings may help guide the ongoing efforts to optimize therapy, in order to overcome the evolutionary potential of CLL that leads to relapse. First, these results support the investigation of ibrutinib therapy in the treatment-naive setting where clonal complexity is expected to be lower^5^. Second, these results add to considerations about early treatment versus a “watch and wait” approach in CLL, as earlier stage disease may harbor less clonal diversity and therefore may have reduced evolutionary potential. Third, the progressive transcriptional changes seen over time suggest that the therapeutic effect of ibrutinib may be extended with longer use by allowing ongoing microenvironmental remodeling. Finally, the estimation of clone sizes at treatment initiation may serve to optimize combination therapy, as therapies combined with ibrutinib would need to specifically target these resistant cell populations.

## Methods

### Patients and samples

Cohort A included 45 of 86 subjects enrolled on a phase 2 study of ibrutinib 420 mg once daily in patients with CLL, based on sample availability. Inclusion criteria included ≥ 65 years of age or CLL with del(17p) or *TP53* mutation at the National Institutes of Health (NIH) Clinical Center (NCT01500733). Cohort B included 16 of 41 enrolled subjects on a phase 2 study of rituximab 375 mg/m^2^ on days 1, 8, 15, and 22, then once every 4 weeks during cycles 2–6 and ibrutinib 420 mg once daily starting on cycle 1 day 2 at the University of Texas MD Anderson Cancer Center (MDACC, NCT01520519). Inclusion criteria included diagnosis of high-risk CLL with either the presence of a 17p deletion or 11q deletion or TP53 mutation or previous treatment with up to three lines of prior therapy. Each clinical trial was approved by the NIH National Heart, Lung, and Blood and the MDACC institutional review board, respectively, and conducted in accordance with the Declaration of Helsinki and the International Conference on Harmonization Guidelines for Good Clinical Practice. Written informed consent was obtained from all participants. Heparinized blood was collected before and after initiation of ibrutinib therapy; peripheral blood mononuclear cells from patient samples were isolated by Ficoll/Hypaque density-gradient centrifugation. Tumor cells were purified by CD19^+^ selection with MACS Cell Separation Columns (Miltenyi Biotec, Cambridge, MA) at 4 °C and stored as pellets at −80 °C. Of note, we have previously shown that during the first year of ibrutinib therapy the proportion of CD19 + non-malignant cells remains low (<5%)^41^, and have confirmed this by flow cytometry of the samples analyzed in the current work.

### Prognostic marker and *IGHV* analysis

Pretreatment evaluation included interphase FISH for common CLL chromosomal abnormalities with Vysis probes (Abbott Molecular, Des Plaines, IL) and sequencing of the *IGHV* gene in tumor cells. *IGHV* sequences were aligned to germline sequences in the international ImMunoGeneTics (IMGT) information system and database tools (IMGT/V-Quest)^42^. As per convention, the *IGHV* somatic mutation status was designated as unmutated if there was ≥98% homology; or as mutated if there was <98% homology to germline sequences^43^.

### Nucleic acid extraction and quality control

Genomic DNA from CLL cells and matched germline DNA (from bone marrow stromal cells or saliva) were extracted per manufacturer’s recommendations (Qiagen, Germantown, MD). The concentrations of tumor and normal DNA were measured using PicoGreen dsDNA Quantitation Reagent (Invitrogen, Carlsbad, CA). A minimum DNA concentration of 60 ng/ml was required for sequencing and each Illumina sequencing library was created using the native DNA. Mass spectrometric fingerprint genotyping of 24 common SNPs (Sequenom, San Diego, CA) was used to confirm the identities of all tumor and normal DNA samples. Standard RNA extraction protocols (RNAeasy kit, Qiagen, Germantown, MD) were used to extract RNA from CLL-B cells.

### Whole-exome sequencing data generation and preprocessing

Samples were sequenced using Agilent SureSelect capture kit and Illumina Rapid Capture Enrichment—37 Mb target kit on Illumina next-generation sequencers. The sequencing data processing pipeline known as “Picard” (http://broadinstitute.github.io/picard/) was used to generate a BAM file for each sample. Picard consists of four steps.

The first step involves alignment to the genome. Here, BWA is used to align the sequence data to the NCBI Human Reference Genome GRCh37/hg19^44^. Within the BAM file, sequence reads are sorted by chromosomal position and unaligned reads passing the Illumina quality filter (PF reads) are also included in the BAM file.

The second step involves base recalibration. Here, each base within a read sequence is assigned a Phred-like quality *Q* score that represents the probability that the base call is erroneous. The *Q* score represents −10*log (probability of error) and is rounded to an integer value. We also used GATK to empirically recalibrate the qualities according to the original *Q* score (generated by the Illumina software), the lane, the read cycle, the tile, the base in question and the preceding base^45^. The original quality scores are also represented by the read-level OQ tag within the BAM file.

The third step involves aggregation of lane and library level data. Here, for each sample, multiple lanes and libraries were aggregated into a single BAM file. Lane-level BAM files were then combined into a single BAM file for each sample. The read group information within these BAM files represent the library and lane information. Information regarding the read groups appears in the BAM header.

The fourth step involves marking of duplicated reads. The MarkDuplicates algorithm from Picard (http://broadinstitute.github.io/picard/) is used here to flag molecular duplicate reads. Pairs of reads in which both ends map to the identical genomic position are considered as arising from the same DNA molecule and thus considered duplicate reads. Only one of these duplicate reads is retained in the BAM file.

Following the generation of BAM files using Picard, Firehose (http://archive.broadinstitute.org/cancer/cga/firehose) was used to analyze the whole-exome sequencing data. Firehose has been developed at the Broad Institute. All tumor-normal pairs were required to pass the Firehose QC pipeline. This QC pipeline involved testing for DNA contamination of a sample from other individuals using the ContEst algorithm, together with cross-checking lane fingerprints.

### Mutation calling

Somatic mutations were identified in targeted exon data using the MuTect algorithm, version 1.1.6^46^. MuTect involves the Bayesian statistical analysis of bases and their qualities to identify candidate somatic mutations at a given genomic locus using tumor and normal BAM files. The ContEst algorithm^47^ was used to estimate the level of cross-contamination which was in turn used to estimate the lowest allelic fraction at which somatic mutations could be detected on a per-sample basis. Candidate indels (small insertions and deletions) were detected using Indelocator (http://archive.broadinstitute.org/cancer/cga/indelocator). All mutations were filtered using a panel of normals filter, which removes mutations commonly seen across a large number of sequenced normal (non-cancer) samples. All paired tumor-normal pairs were run through deTiN, a Bayesian method to estimate the contamination of tumor DNA in the normal sample, and keep mutations which would otherwise have been removed by germline filters (manuscript in preparation, Taylor-Weiner et al.) Furthermore, manual review of somatic mutations from their respective BAM files using the Integrative Genomics Viewer (IGV)^48^ was also used to filter mutations. For each patient, the union of all point mutations and indels from every sample was created. Then, the mutant and reference allele counts of every mutation in this union were measured in each sample using samtools in a process called force-calling^49^.

### Somatic copy number alteration identification

The ReCapSeq tool (version 34)^50^ was used to estimate the coverage profile for each tumor sample. In brief, this tool first normalizes read coverage for each target segment using the total number of aligned reads. The coverage for every segment is then normalized using tangent normalization against the coverage present across a Panel of Normals that have been sequenced using the same target regions. The circular binary segmentation algorithm^51^ is then used to merge target regions so as to form segments that correspond to the same copy number event. The allelic copy ratio in each tumor sample was then estimated by measuring the allelic fraction of germline heterozygous SNPs. These allelic copy ratio estimates were then combined with the observed copy ratio of each segment.

### Calculation of cancer cell fraction and clustering

ABSOLUTE^24^ was performed to assess purity and overall ploidy of each sample, as well as the cancer cell fraction (CCF) of each mutation (percentage of tumor cells harboring mutation in the sample). The ABSOLUTE algorithm requires two inputs, an input maf (mutation annotation file), and segmented allelic specific copy ratio file (allelic seg file). The allelic seg file was produced as described in the previous section. The input maf file passed in to ABSOLUTE for each sample contained all of the force-called mutation calls. This made it possible to estimate the CCF of mutations which were not originally detected in a given sample, but detected in other samples belonging to that patient. ABSOLUTE produces an array of possible purity/ploidy combinations, ordered by their relative likelihoods. The final purity and ploidy of each sample were chosen by manual review of these possible solutions.

To estimate the number of clusters and subsequent clonal structure in each individual, we further developed an approach previously published by our group^52^, and improved and extended the method to incorporate analysis of a large number of samples from a single patient. In brief, the method uses a Bayesian clustering framework based on a Dirichlet Process^53^, where the parameter defining the number of clusters is varied during the process and inferred over many MCMC iterations.

For each individual in the cohort (*n* = 61), the two clusters with maximal negative and positive change between baseline and latest post-treatment samples were identified. For each of these clusters the posterior probability density of the two-dimensional cluster CCF distribution lying within ±10% of the diagonal was quantified to evaluate the null assumption that the cluster CCF was similar between timepoints. These probabilities were submitted to a Benjamini–Hochberg FDR procedure to account for multiple hypotheses testing, and the null was rejected if the adjusted *P* value was <0.1.

### Clonal kinetics analysis and growth rate inference

An MCMC (Monte-Carlo Markov Chain) algorithm was run on the final clustering results of the four individuals shown in Figs. 5, 6, to determine growth and death rates in each clone which had a maximal cluster CCF of at least 0.1 in at least one time point. CCF values were converted to absolute cell numbers by multiplying the ALC by the blood volume in microliters, sample purity and clone-specific CCF. Phylogenetic trees and clusters were fixed at the beginning of the MCMC iterations, with a total of 10,000 MCMC iterations run per individual. Within each iteration, mutations were assigned randomly to a clone from a multinomial random distribution based on the likelihood of each mutation belonging to that clone. Finally, the growth rates were calculated at the end of the iteration by fitting the clustered CCFs of each clone at each time point to an exponential curve. For the relapse case A11, as the cluster only contained the single *CDIPT* event it was consistently merged with other neighboring clusters during MCMC iterations. Thus, the exponential growth curve for the clone containing a single *CDIPT* deletion was fitted using a linear regression of the CCF point estimates for this subclone. Phylogenies of subclones within each case were assigned based on the following constraints: (i) parent-progeny relationships were assigned only if the parent clone had a higher cancer cell fraction than the progeny clone in all samples, and sibling relationships were enforced by the constraint of sibling clones having a summed cancer cell fraction of no more than 1 at any timepoint. When more than one phylogeny resulted from the application of these constraints, the phylogenetic relationship that maximized the goodness of fit for the exponential growth model of each clone was chosen.

### Targeted sequencing of *BTK* and *PLCG2* genes

Exon 15 of *BTK* and exons 19, 20, and 24 of *PLCG2* were amplified using custom oligonucleotides and analyzed by bidirectional Sanger sequencing. To increase the limit of detection for hotspot mutations, mutant alleles were preferentially amplified by wild-type blocking polymerase chain reactions followed by hybrid-capture based next-generation sequencing with custom SureSelect QXT Target Enrichment (Agilent; La Jolla, CA) or Nextera Rapid Capture (Illumina; San Diego, CA) panels that include *BTK* and *PLCG2* genes (NeoGenomics Laboratories, Irvine, CA).

### RNA-sequencing analysis

A cDNA library was prepared from poly-A selected RNA and sequenced on an Illumina platform. The total counts of reads across samples were normalized, and log_2_ transformed counts data were used for downstream analysis. Subject effect and time effect of treatment of the samples were qualitatively assessed by the first few principal components of the data. A two-way ANOVA model was then used to account for subject effect and time point effect for each gene. Genes with 2 fold-change in expression on treatment compared to baseline and with a false-discovery rate less than 10% were considered differentially expressed and selected for further investigation. Overrepresentation of experimentally derived gene sets in the list of selected genes was estimated by the *P* values (FDR adjusted *P* values) calculated from hypergeometric distribution. Under-enrichment or over-enrichment scores were calculated based on the cumulative distribution function of the hypergeometric distribution. The Graeber Lab CDF calculator^54^ (UCLA, CA), JMP 12 (Cary, NC) and R software^55^ were used in data analysis. Gene sets with a minimum of five differentially expressed genes, *P* < 0.05 and a FDR *q* < 0.1 were considered overrepresented. The mean change in gene expression was calculated by averaging the change across all differentially expressed genes per patient at each time point for each overrepresented gene set.

### General statistical considerations

Associations between clonal evolution, progressive disease and clinical features were assessed by the Wilcoxon rank-sum test or Fisher exact test, as appropriate. The Kaplan–Meier method was used to estimate time-to-progression and differences between groups were compared using the log-rank test. Associations between patient groups were determined by unpaired Student's *t* test, while associations across gene sets overtime was determined by paired Student's *t* test.

### Data availability

The sequence data have been deposited in dbGaP under the accession code 26784.

## Electronic supplementary material

Supplementary Information

Description of Additional Supplementary Files

Supplementary Data 1

Supplementary Data 2

Supplementary Data 3

Supplementary Data 4

Supplementary Data 5

Supplementary Data 6

## References

[1] Schuh A (2012). Monitoring chronic lymphocytic leukemia progression by whole genome sequencing reveals heterogeneous clonal evolution patterns. Blood.

[2] Fabbri G (2013). Genetic lesions associated with chronic lymphocytic leukemia transformation to Richter syndrome. J. Exp. Med..

[3] Landau DA, Carter SL, Getz G, Wu CJ (2014). Clonal evolution in hematological malignancies and therapeutic implications. Leukemia.

[4] Landau DA, Wu CJ (2013). Chronic lymphocytic leukemia: molecular heterogeneity revealed by high-throughput genomics. Genome Med..

[5] Landau DA (2013). Evolution and impact of subclonal mutations in chronic lymphocytic leukemia. Cell.

[6] Landau DA (2015). Mutations driving CLL and their evolution in progression and relapse. Nature.

[7] Fabbri G, Dalla-Favera R (2016). The molecular pathogenesis of chronic lymphocytic leukaemia. Nat. Rev. Cancer.

[8] Byrd JC (2013). Targeting BTK with ibrutinib in relapsed chronic lymphocytic leukemia. N. Engl. J. Med..

[9] Farooqui MZ (2015). Ibrutinib for previously untreated and relapsed or refractory chronic lymphocytic leukaemia with TP53 aberrations: a phase 2, single-arm trial. Lancet Oncol..

[10] Byrd JC (2014). Ibrutinib versus ofatumumab in previously treated chronic lymphoid leukemia. N. Engl. J. Med..

[11] Byrd JC (2015). Three-year follow-up of treatment-naive and previously treated patients with CLL and SLL receiving single-agent ibrutinib. Blood.

[12] O’Brien S (2014). Ibrutinib as initial therapy for elderly patients with chronic lymphocytic leukaemia or small lymphocytic lymphoma: an open-label, multicentre, phase 1b/2 trial. Lancet Oncol..

[13] Burger JA (2015). Ibrutinib as initial therapy for patients with chronic lymphocytic leukemia. N. Engl. J. Med..

[14] Woyach JA (2014). Resistance mechanisms for the Bruton’s tyrosine kinase inhibitor ibrutinib. N. Engl. J. Med..

[15] Furman RR (2014). Ibrutinib resistance in chronic lymphocytic leukemia. N. Engl. J. Med..

[16] Burger JA (2016). Clonal evolution in patients with chronic lymphocytic leukaemia developing resistance to BTK inhibition. Nat. Commun..

[17] Ahn IE (2017). Clonal evolution leading to ibrutinib resistance in chronic lymphocytic leukemia. Blood.

[18] Scherer F (2016). Distinct biological subtypes and patterns of genome evolution in lymphoma revealed by circulating tumor DNA. Sci. Transl. Med..

[19] Ojamies PN (2016). Monitoring therapy responses at the leukemic subclone level by ultra-deep amplicon resequencing in acute myeloid leukemia. Leukemia.

[20] Adzhubei IA (2010). A method and server for predicting damaging missense mutations. Nat. Methods.

[21] Forbes SA (2015). COSMIC: exploring the world’s knowledge of somatic mutations in human cancer. Nucleic Acids Res..

[22] Wilson WH (2015). Targeting B cell receptor signaling with ibrutinib in diffuse large B cell lymphoma. Nat. Med..

[23] Davis RE (2010). Chronic active B-cell-receptor signalling in diffuse large B-cell lymphoma. Nature.

[24] Carter SL (2012). Absolute quantification of somatic DNA alterations in human cancer. Nat. Biotechnol..

[25] Burger JA (2014). Safety and activity of ibrutinib plus rituximab for patients with high-risk chronic lymphocytic leukaemia: a single-arm, phase 2 study. Lancet Oncol..

[26] Valsecchi R (2016). HIF-1alpha regulates the interaction of chronic lymphocytic leukemia cells with the tumor microenvironment. Blood.

[27] Nishida M (2003). Amplification of receptor signalling by Ca^2+^ entry-mediated translocation and activation of PLCgamma2 in B lymphocytes. Embo. J..

[28] Kroczek C (2010). Swiprosin-1/EFhd2 controls B cell receptor signaling through the assembly of the B cell receptor, Syk, and phospholipase C gamma2 in membrane rafts. J. Immunol..

[29] Maddocks KJ (2015). Etiology of ibrutinib therapy discontinuation and outcomes in patients with chronic lymphocytic leukemia. JAMA Oncol..

[30] Chan W, Schaffer TB, Pomerantz JL (2013). A quantitative signaling screen identifies CARD11 mutations in the CARD and LATCH domains that induce Bcl10 ubiquitination and human lymphoma cell survival. Mol. Cell Biol..

[31] Pasqualucci L (2011). Analysis of the coding genome of diffuse large B-cell lymphoma. Nat. Genet..

[32] Sauer K, Cooke MP (2010). Regulation of immune cell development through soluble inositol-1,3,4,5-tetrakisphosphate. Nat. Rev. Immunol..

[33] Messmer BT (2005). In vivo measurements document the dynamic cellular kinetics of chronic lymphocytic leukemia B cells. J. Clin. Invest..

[34] Niemann CU (2016). Disruption of in vivo chronic lymphocytic leukemia tumor-microenvironment interactions by ibrutinib--findings from an investigator-initiated phase II Study. Clin. Cancer Res..

[35] Dubovsky JA (2013). Ibrutinib is an irreversible molecular inhibitor of ITK driving a Th1-selective pressure in T lymphocytes. Blood.

[36] Bozic I, Nowak MA (2014). Timing and heterogeneity of mutations associated with drug resistance in metastatic cancers. Proc. Natl Acad. Sci. USA.

[37] Bozic I (2013). Evolutionary dynamics of cancer in response to targeted combination therapy. Elife.

[38] Mareschal S (2016). Whole exome sequencing of relapsed/refractory patients expands the repertoire of somatic mutations in diffuse large B-cell lymphoma. Genes Chromosomes Cancer.

[39] Dubois S (2016). Next-generation sequencing in diffuse large B-cell lymphoma highlights molecular divergence and therapeutic opportunities: a LYSA Study. Clin. Cancer Res..

[40] Siegemund S (2015). IP3 3-Kinase B suppresses B cell lymphoma by antagonizing PI3K/mTOR in B cells. J. Immunol..

[41] Sun C (2015). Partial reconstitution of humoral immunity and fewer infections in patients with chronic lymphocytic leukemia treated with ibrutinib. Blood.

[42] Lefranc MP (2015). IMGT(R), the international ImMunoGeneTics information system(R) 25 years on. Nucleic Acids Res..

[43] Hamblin TJ, Davis Z, Gardiner A, Oscier DG, Stevenson FK (1999). Unmutated Ig V(H) genes are associated with a more aggressive form of chronic lymphocytic leukemia. Blood.

[44] Li H, Durbin R (2009). Fast and accurate short read alignment with Burrows-Wheeler transform. Bioinformatics.

[45] McKenna A (2010). The Genome Analysis Toolkit: a MapReduce framework for analyzing next-generation DNA sequencing data. Genome Res..

[46] Cibulskis K (2013). Sensitive detection of somatic point mutations in impure and heterogeneous cancer samples. Nat. Biotechnol..

[47] Cibulskis K (2011). ContEst: estimating cross-contamination of human samples in next-generation sequencing data. Bioinformatics.

[48] Robinson JT (2011). Integrative genomics viewer. Nat. Biotechnol..

[49] Li H (2009). The Sequence Alignment/Map format and SAMtools. Bioinformatics.

[50] Lichtenstein, L., Woolf, B., MacBeth, A., Birsoy, O. & Lennon, N. in *AACR 107th Annual Meeting* (New Orleans, LA, 2016).

[51] Olshen AB, Venkatraman ES, Lucito R, Wigler M (2004). Circular binary segmentation for the analysis of array-based DNA copy number data. Biostatistics.

[52] Brastianos PK (2015). Genomic characterization of brain metastases reveals branched evolution and potential therapeutic targets. Cancer Discov..

[53] Escobar MD, West M (1995). Bayesian density estimation and inference using mixtures. J. Am. Stat. Assoc..

[54] Plaisier SB, Taschereau R, Wong JA, Graeber TG (2010). Rank-rank hypergeometric overlap: identification of statistically significant overlap between gene-expression signatures. Nucleic Acids Res.

[55] R Development Core Team. *R: A Language and Environment for Statistical Computing. R Foundation for Statistical Computing* (R Development Core Team, Vienna, Austria, 2010).

