# Supplementary material for: The evolutionary landscape of chronic lymphocytic leukemia treated with ibrutinib targeted therapy
Source: Nat Commun. 2017 Dec 19;8:2185. doi: 10.1038/s41467-017-02329-y (PMC5736707; doi:10.1038/s41467-017-02329-y)
Supplement: Supplementary file 1 — Supplementary Information [file 41467_2017_2329_MOESM1_ESM.pdf]

Supplementary Information

Supplementary Figures

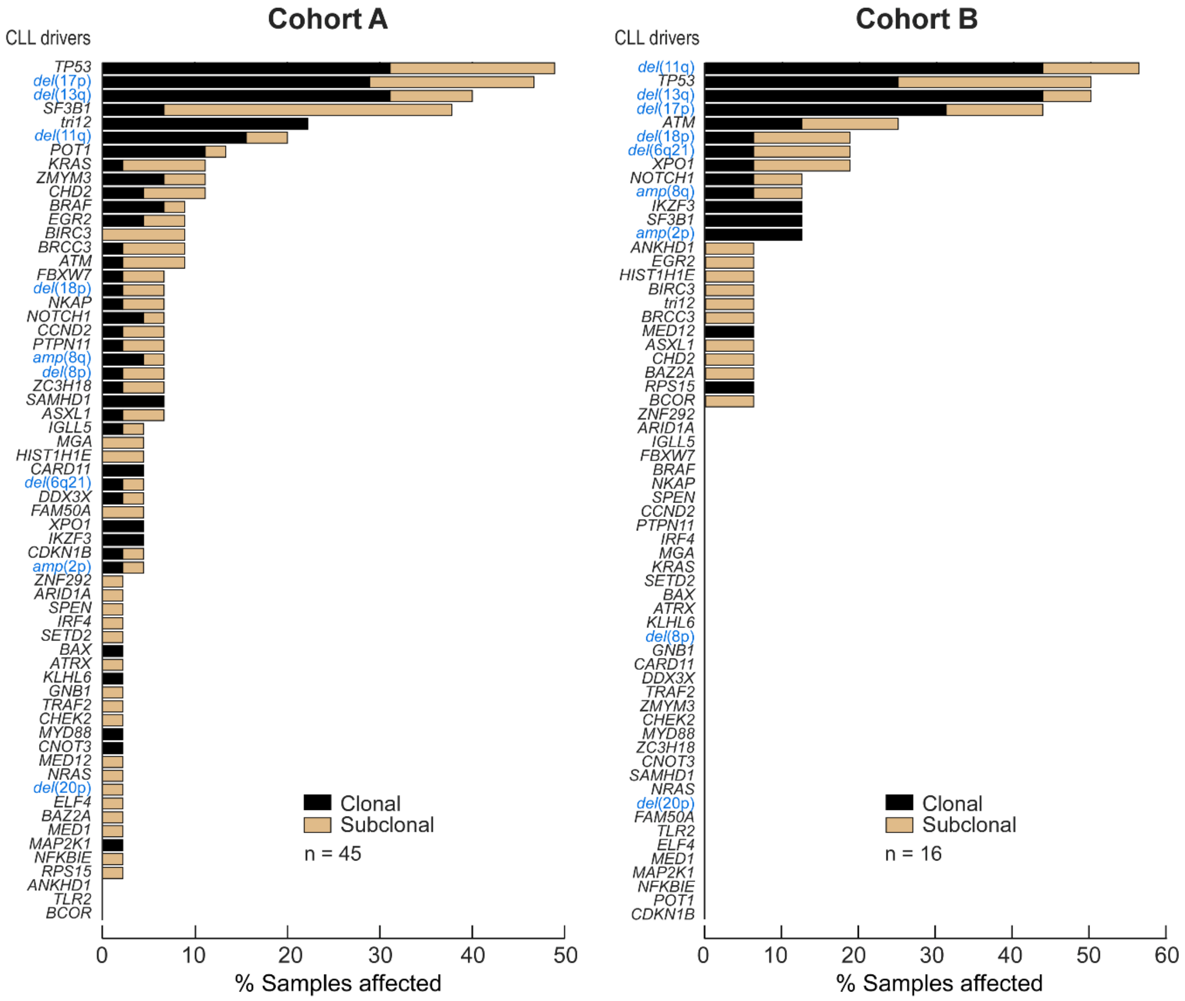

Supplementary Figure 1. Clonal and subclonal status of putative driver mutations in cohorts A and B**.** Distribution of clonal (black) and subclonal (tan) putative driver mutations and copy number alterations (blue font) across the two patient cohorts.

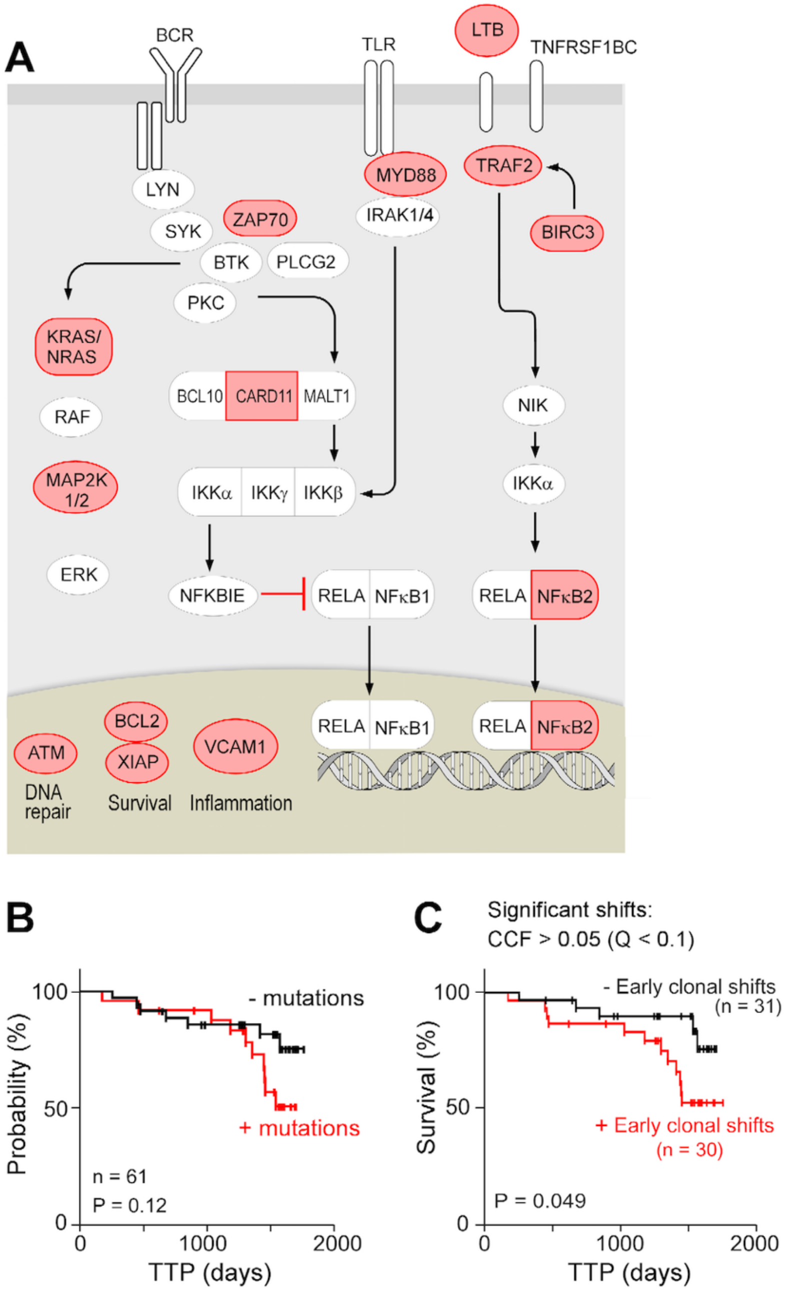

Supplementary Figure 2. Somatic mutations in the BCR and NF-κB pathways identified in Cohorts A and B, and time-to-progression analysis in relation to presence of these mutations. (**a**) Somatic mutations in members of the BCR and NF-κB pathways (pink), identified by analysis of WES of 61 pretreatment CLL samples. (**b**) Kaplan-Meier plot of time-to-progression separated by the presence (red) or absence (black) of BCR and NF-κB pathway mutations. (**c**) Kaplan-Meier plot of time to progression separated by the presence (red) or absence (black) of early clonal shifts (CCF >0.05 with Q value <0.1).

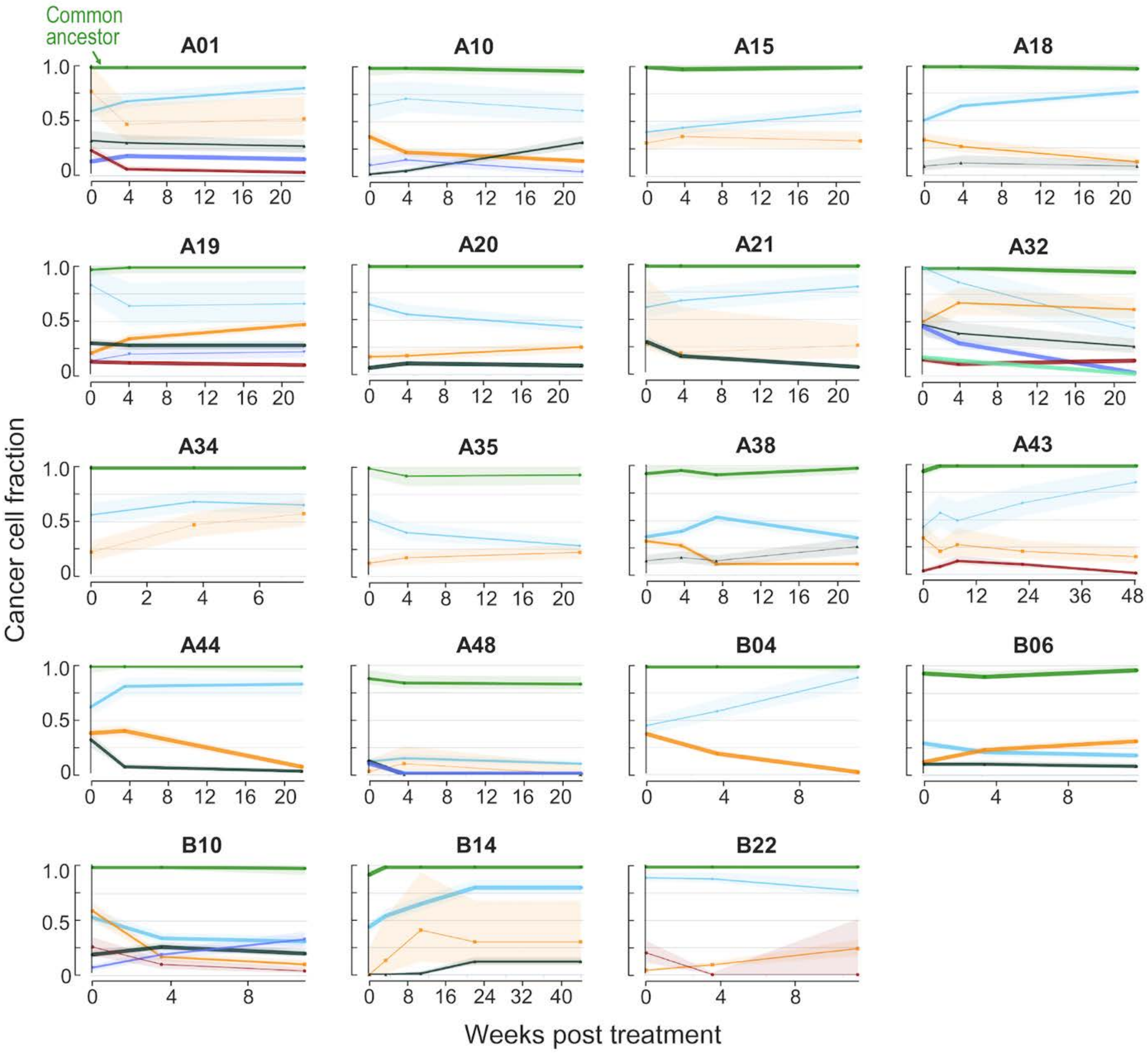

Supplementary Figure 3. Clonal change in CCF over time in 19 CLLs treated with ibrutinib *with* statistically significant early clonal shifts. The CCF mode is calculated per clone/timepoint based on SNV data. The shaded area represents a 95% CI.

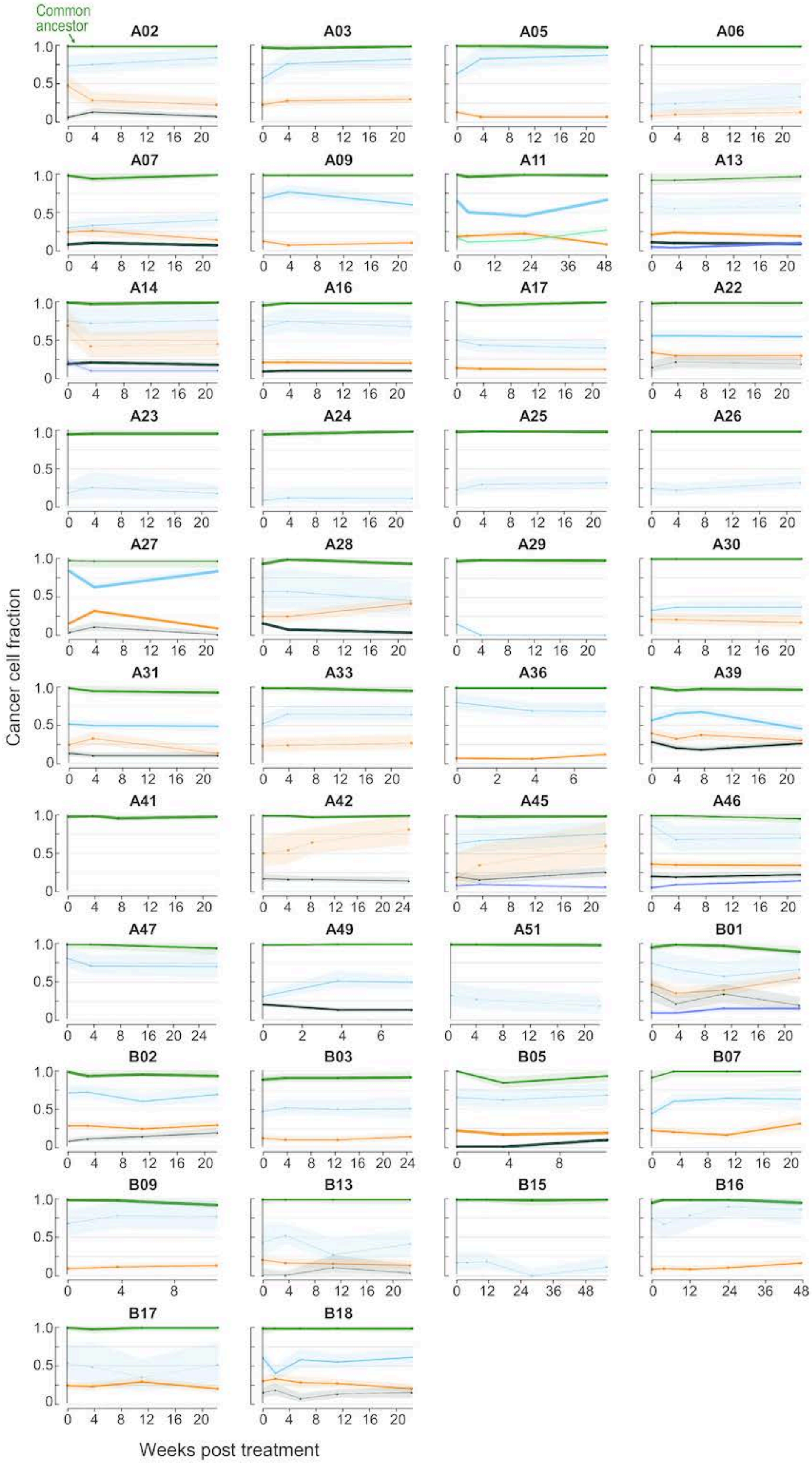
Common ancestor
A02
A03
A05
A06
A07
A09
A11
A13
A14
A16
A17
A22
A23
A24
A25
A26
A27
A28
A29
A30
A31
A33
A36
A39
A41
A42
A45
A46
A47
A49
A51
B01
B02
B03
B05
B07
B09
B13
B15
B16
B17
B18
Cancer cell fraction
Weeks post treatment

Supplementary Figure 4. Clonal change in CCF over time in 42 CLLs treated with ibrutinib *without* statistically significant early clonal shifts. The CCF mode is calculated per clone/timepoint based on SNV data. The shaded area represents a 95% CI. We note that this group contains cases which appear to have some shifts that either did not reach statistical significance due to measurement uncertainty (wide CI, e.g., A42) or due to fluctuations over the treatment period (e.g., A27) that did not result in a significant change comparing the first and last observation.

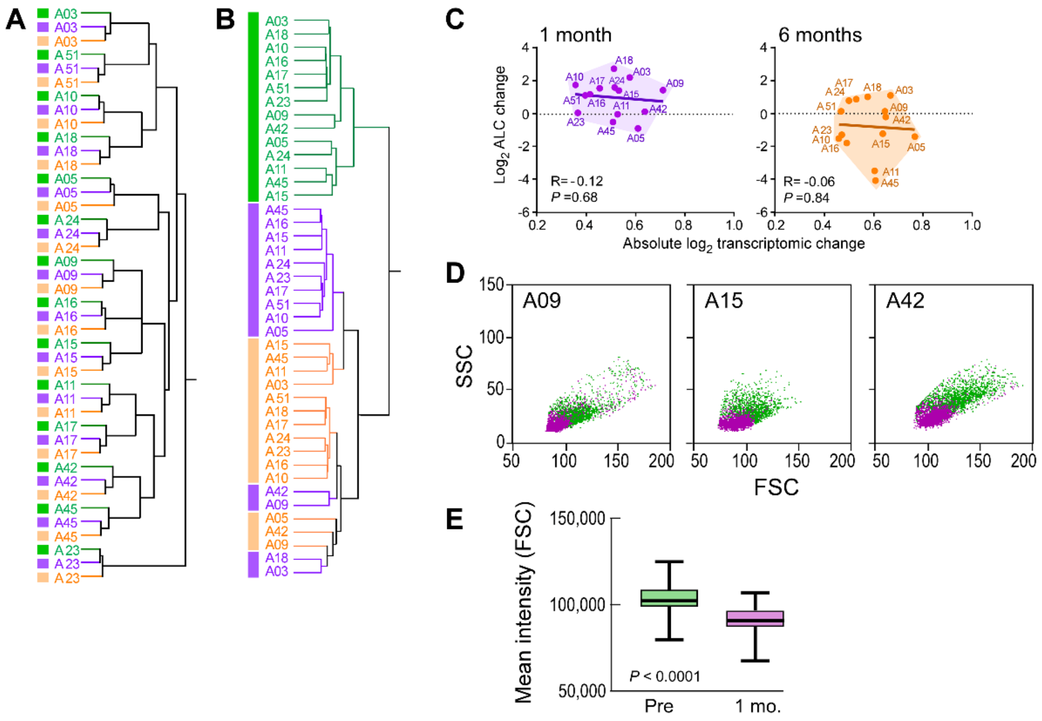

Supplementary Figure 5. Transcriptomic changes of Cohort A samples on ibrutinib treatment. (**a**) Hierarchial clustering of samples based on principal component analysis (PCA) across 14 patients and three time points (pre [green], 1 month post-treatment initiation [purple] and 6 months post-treatment initiation [orange]). Sample names are indicated (e.g., 'A15'). (**b**) Hierarchial clustering of samples by PCA corrected for inter-patient variability. (**c**) Lack of correlation between change in absolute lymphocyte count to change in gene expression across all coding genes, following 1 month (left, R=-0.12; P=0.68) and 6 months (right, R=-0.06; P=0.84) on therapy. R- and P-values determined by Pearson correlation. (**d**) Representative FACS scatter plots with overlay of pre (green) vs 1 month post (purple) cells, showing decrease size with treatment based on side (SSC) and forward (FSC) scatter, gated on live cells. Analyzed on a FACS Canto II flow cytometer (BD Biosciences) using FACS-DIVA 6.1.1 and FlowJo (Version 10, TreeStar). (**e**) Change in mean FSC (+/- standard error of mean) at pre-treatment (green) and 1 month post treatment initiation (purple, n=9). P-values determined by a paired student t-test.

Supplementary Tables

Supplementary Table 1: Cohorts

	N (%) or Median (range)	Cohort A (WES)	Cohort A (RNA-seq)	Cohort B (WES)
Patient number		45	14	16
Age	65 (33-85)	66 (33-85)		63 (35-78)
Male gender	38 (62%)	27 (60%)		11 (69%)
Rai stage				
0	1 (2%)	0 (0%)		1 (6%)
1	12 (20%)	9 (20%)		3 (19%)
2	10 (16%)	8 (18%)		2 (13%)
3	10 (16%)	8 (18%)		2 (13%)
4	28 (46%)	20 (44%)		8 (50%)
Unmutated IGHV	39 (64%)	27 (60%)	9 (64%)	12 (75%)
FISH				
Deletion 17p	32 (52%)	25 (56%)	8 (57%)	7 (44%)
Deletion 11q	18 (30%)	9 (20%)		9 (56%)
Trisomy 12	8 (13%)	7 (16%)		1 (6%)
Deletion 13q	33 (54%)	22 (49%)		11 (69%)
Relapsed/refractory	34 (56%)	19 (42%)	8 (57%)	15 (94%)

Supplementary Table 2: Progressive disease cases

Patient ID	Age	Sex	FISH	IGHV	Treatment Status[1]	Treatment	Best Response[2]	Time to Progression (Months)	Pathology at Progression
MDAC-0022	35	F	11q del	U	RR	Ib + R	PR	6	CLL
NHLBI-0042	66	M	Del 17p, Del 13q	U	RR	Ib	SD	9	Diffuse large B-cell lymphoma
NHLBI-0003	66	F	Del 17p	U	RR	Ib	PRL	15	Hodgkin-like cells mixed with CLL
NHLBI-0034	56	M	Del 17p	U	TN	Ib	PR	15	Diffuse large B-cell lymphoma
MDAC-0006	73	F	17p del, 13q del	U	RR	Ib + R	PR	16	CLL
MDAC-0018	78	F	13q del, 17p del	U	RR	Ib + R	PR	23	CLL
NHLBI-0006	77	M	Tri 12, Del 13q	U	RR	Ib	PR	28	CLL
MDAC-0010	58	M	11q del, 17p del	U	RR	Ib + R	PR	34	CLL
NHLBI-0011	62	F	Del 17p	U	RR	Ib	PR	39	CLL
NHLBI-0043	64	F	Del 17p	M	TN	Ib	PR	43	CLL
NHLBI-0019	59	M	Normal	M	RR	Ib	PR	45	CLL
MDAC-0014	65	M	17p del, 13q del	NA	RR	Ib + R	PR	47	CLL
NHLBI-0005	65	M	Tri 12	U	RR	Ib	PR	48	CLL
NHLBI-0020	66	F	Del 17p	M	RR	Ib	CR	48	CLL
NHLBI-0021	61	F	Normal	U	RR	Ib	CR	48	CLL
MDAC-0003	56	M	11q del, 13q del	NA	RR	Ib + R	PRL	51	CLL
MDAC-0015	45	M	13q del	NA	RR	Ib + R	PR	52	CLL

[1]RR = relapsed/refractory, TN = treatment naïve
[2]PR = partial response, SD = stable disease, PRL = partial response with lymphocytosis, CR = complete response

Supplementary Table 3: Targeted BTK and PLCG2 sequencing

Patient ID	Gene	AA change	MT codon	WT codon	VAF (%)
A03[2]	None detected				
A05[2]	BTK	C481S	TGC	TCC	2.2
A06[2]	BTK	C481S	TGC	TCC	78.2
	PLCG2	R665W	CGG	TGG	0.26
	PLCG2	S707Y	TCC	TAC	0.17
	PLCG2	L845F	TTA	TTT	4.7
A11[2]	None detected				
A19	BTK	C481S	TGC	TCC	N/A[1]
A20	BTK	C481S	TGC	TCC	43.7
A21	PLCG2	R665W	CGG	TGG	N/A[1]
A34[2]	PLCG2	R665W	CGG	TGG	0.11
A42[2]	None detected				
A43[2]	None detected				

[1]VAF not available as only Sanger sequencing performed
[2]Previously reported in Ahn et al.
