# Supplementary material for: The evolutionary landscape of chronic lymphocytic leukemia treated with ibrutinib targeted therapy
Source: Nat Commun. 2017 Dec 19;8:2185. doi: 10.1038/s41467-017-02329-y (PMC5736707; doi:10.1038/s41467-017-02329-y)
Supplement: Supplementary file 2 — Description of Additional Supplementary Files [file 41467_2017_2329_MOESM2_ESM.pdf]

Description of Supplementary Files

File Name: Supplementary Data 1
Description: Sequencing metrics of the WES data generated from Cohorts A and B

File Name: Supplementary Data 2
Description: Summary of the CLL driver mutations identified in the baseline pretreatment CLL samples from Cohorts A and B

File Name: Supplementary Data 3
Description: Summary of the CLL copy number alterations identified in the baseline preteatment CLL samples from Cohorts A and B

File Name: Supplementary Data 4
Description: Summary of the mutations in the BCR and NF-kB pathways identified in the baseline pretreatment CLL samples from Cohorts A and B

File Name: Supplementary Data 5
Description: Differentially expressed genes when comparing pre-treatment RNA sequencing with RNA sequencing at 1 and 6 months.

File Name: Supplementary Data 6
Description: Differentially expressed gene set enrichment when comparing pre-treatment RNA sequencing with RNA sequencing at 1 and 6 months.
